# Outside the Military “Bubble”: Life After Service for UK Ex-armed Forces Personnel

**DOI:** 10.3389/fpubh.2020.00050

**Published:** 2020-03-03

**Authors:** Kim Gordon, Karen Burnell, Clare Wilson

**Affiliations:** ^1^School of Life Sciences and Education, Clinical Psychology, Staffordshire University, Stoke-on-Trent, United Kingdom; ^2^School of Sport, Health and Social Sciences, Solent University, Southampton, United Kingdom; ^3^Department of Psychology, University of Portsmouth, Portsmouth, United Kingdom

**Keywords:** British ex-Armed Forces, veterans, transition, well-being, interventions, mental health service access, trauma

## Abstract

Military personnel who have seen active service can be affected by their experiences. Much of the literature on the mental and physical health battles faced by men and women who leave the Armed Forces is dominated by research in the United States (US) (1), and is particularly focused on exposure to deployment, combat conditions, and effects on mental health. Research in the United Kingdom (UK) tends to focus on depression or alcohol misuse and the impact these issues have on currently serving personnel. This study aimed to present UK veterans' first-hand experiences related to military service, access to and use of mental healthcare and interventions, and the impact of transition on the military family. Semi-structured interviews explored experiences of 30 participants (27 male, 3 female). Participants ranged in age from 26 to 92 years (*M* = *53.33*), and across multiple war cohorts (from WWII to Iraq and Afghanistan). Data were analyzed using Thematic Analysis and Narrative Analysis. Findings show meaning-making from experiences of transition across veteran cohorts. Main themes were reasons for leaving Armed Forces, life outside the military, and mental health concerns after service. Subordinate themes additionally focused on evaluation of identity and mental health service provision. Future clinical research should include the experiences of UK serving personnel and the effects of pre-and post-military adversity, alongside the impact of deployment experiences. Interventions designed to address transition into life after service are discussed.

## Introduction

The effects of service on military personnel is founded on historical research in the United States (US), particularly on Vietnam veterans (1, 2). Current research tends to begin with examinations of the effects of combat exposure and issues arising from mental and physical health battles faced by ex-service personnel, such as depression or alcohol misuse (3). Examinations of the impact these issues have on currently serving personnel, look at a decrease in quality of life and the use of treatment interventions as a result of service exposure (4), as well as barriers to the access of those interventions (3). Although much was learned about mental health issues in US Vietnam veterans and what challenges present-day veterans face, research on US veterans and the mental and physical effects of service in the most recent conflicts, have presented differently or less frequently in UK veterans (5).

Studies of UK veterans and help-seeking behavior among ex-service personnel are concepts explored in the context of ideas around stigma around mental health, transition, and adaption experiences of formerly serving personnel (6) and further studies of the effects of their military experience was recommended (7, 8). Military service personnel have different responses to combat exposure and there are differences between nations, military cohorts, and across conflict conditions. After the most recent conflicts in the Balkans and during the Middle East, 2005 marked a change in the need for specific research on the negative effects of service on members of the UK Armed Forces during and after service (5, 9). Research of potential pre-service impact on veterans' well-being and well-being of the family appear less frequently (10, 11).

Factors that may prevent personnel from seeking help as well as predict later mental health issues in personnel, was also of interest to the UK military (3). In 2007, childhood adversity was examined to determine whether issues that preceded joining the Armed Forces, mediated psychological trauma in UK combat servicemen (10). In October 2015, the MOD Armed Forces Covenant stressed the importance of combined agencies working to ensure ex-service personnel and their families were not “disadvantaged” by their service to country (7). Memories of events during service can return in later life if individuals do not make sense of experience and come to terms with trauma. For instance, traumatic memories become harder to reconcile as a direct result of cognitive, physical and social age-related changes (12). More detailed accounts of experience may help with understanding how veterans perceive and integrate the influence of pre-service events and quality of life, for instance (13, 14). As countries have and continue to engage in various types of warfare, the interest in and education about veterans and their physical and psychological well-being continues to evolve (15–18) and as the characteristics and needs of veterans have changed over time, knowledge about this population across the lifespan (and the social network around UK veterans) must evolve also. Veterans' experiences provide a framework for questions of what military identity is in the context of transition, what barriers exist to veterans wanting support for psychosocial challenges, and what adaption means for veterans and their families. Accounts of experience are impacted by narratives linked to concepts ranging from pre-service childhood adversity and mental health changes after deployment, for example. The aims of this study, therefore, are to present the specificity of UK Armed Forces veterans' experiences in their own words and across war cohorts: an approach to understanding perceptions and experiences of transition related to pre-service, military, and post-service, and an area under-researched, distinct from previous research literature in military psychology (US Vietnam veterans for example) and from diverse participant data (19).

Qualitative research methods for analyzing narratives of participants were used to address the study objectives. The analysis of narratives considered the physiological, psychological and social aspects of well-being where all are contributing factors to the individual narrative, as well as the barriers to care that military veterans perceived to exist (20). For an in-depth analysis of participant experiences of mental health, interviewing ex-service personnel provided an opportunity to explore how participants make sense of pre-service experiences, service events and related post-service lives.

## Materials and Methods

### Sample

Participants make sense of events and provide detailed context for behavior that may not be captured by structured questionnaires (21, 22). Essential life stories may assist researchers to assess and understand essential aspects of a person's behavior (23). In-depth analysis of participants' storied accounts, then, could provide new insight about what life events mean to them from the beginning, through to the middle of life, and up to the present. This means including an exploration of experiences that exist outside research dominated by service- and combat-related foci and adding to knowledge about the psychosocial needs of veterans (as well as their social circle) from their viewpoint. As a result, 30 ex-service personnel (27 males, 3 females including 1 self-identified as female) from the British Armed Forces between the ages of 26–92, were interviewed about their experiences after leaving the Armed Forces. Interviews took place nationwide between September 2013 and October 2014 and participants had served with the British Army (including Territorial Army WWII), Royal Navy, Royal Marines and Royal Air Force (participant demographics are included in Table 1). In the United Kingdom, the term *veteran* is attributed to any individual who has served for a minimum of 1 day in the Armed Forces.

**Table 1:** Participant demographics pre-service and service history.

**Participant Alias**	**Age at interview**	**Year joined, age**	**Left service**	**Length of service**	**War cohort-era**	**Service corp**	**Pre-service experiences**
Sandy	65	1952, 15	1964	12	Post-WWII	Navy	Homesick when joining. Close to mother and father, communicated with parents while stationed abroad. Served post WWII.
Pete	50	1981, 18	1986	5	Post-Falklands	RAF	Abusive father, witnessed violence toward mother, raised in Northern Ireland social environment of hostility, bullying in community
Terry	38	1991, 17	2006	15	NI, Balkans, Afghanistan x2, Iraq, Kosovo, Macedonia, Sierra Leone	Paras, Royal Marines Commando	Raised by maternal grandmother, little or no contact with mother/father. Married and separated from wife at time of interview and living in temporary housing. Received treatment for PTSD.
Betty	41	1997, 25	2003	6	Afghanistan non-combat	Army (3 years Officer Training Corp before Regular Army)	Recalls content family life and childhood. Followed in footsteps of father in career. Wanted but failed to have a family post-service. Sought marriage counseling.
Nicholas	92	1939, 18	1945	6	WWII	Army	Father WWI suffered mental health. Poverty, family separated, left school to work. Joined Territorial Army. Received surgery for a back injury and was medical discharged from the Artillery division.
Freddie	35	1994, 16	2007	13	Afghanistan	Royal Marines Commando	History of bullying in school. May have experienced childhood assault. Medical discharge for a persistent back injury sustained after a fall. He received treatment for PTSD and is accompanied by a service dog when in public spaces.
Derrick	90	1939, 16	1945	6	WWII	Army	Did not want contact with family after leaving Army. Did not join a veterans' organization. Has not received a hearing aid but has no mental health needs.
Jack	56	1974, 16	1995	21	Falklands, Northern Ireland, Europe, Asia, Central America	Royal Marines Commando	Recalls good childhood experiences growing up in rural town. Divorced after returning from the Falklands. Remarried after deployment to Northern Ireland and received a medical discharge 21 years after service. Received medical and therapeutic treatment for PTSD.
Martin	55	1978, 20	1983	5	Falklands, Northern Ireland	Royal Marines Commando	Recalls good childhood experiences. Deployed to the Falklands and deployed to Northern Ireland shortly after. Received medical discharge and received medical and therapeutic treatment for PTSD, health issues. Separated from wife and seeking custody.
Will	86	1945, 17	1949	4	WWII	RAF	Sent to live with relatives by mother. No positive childhood memories of mother, but idolized father. Sent to work-house, left dockyard work to enlist. Left the Army as a Staff Sergeant. He was deployed to Palestine shortly after end WWII after being decommissioned. Sought out mother who refused contact.
Curtis	47	1990, 23	2013	23	Iraq	Army	Absent of role models and were not “tactile” or emotionally connected family (p. 35). Did not view family positively before or after joining Armed Forces. Academic achievements combined with the lack of a relationship to his family, were motivations behind his joining.
Nigel	52	1980, 18	1989	9	Post-Falklands, Northern Ireland	Navy/Royal Marines Commando	Recalls good childhood experiences, but distant from family after joining Navy. Trained as medic, then joined Commandos. Treated wounded Falklands servicemen, but not deployed. No medical or psychological treatment post-service.
Matty	71	1958, 15	1983	25	Northern Ireland, Europe, Global	Army	Recalls positive family relationship. Joined Army and served in Germany and Northern Ireland during the Troubles (in 1971). Retired voluntarily to work in the public sector after 25 years with no medical or psychological needs.
Paul	62	1967, 15.5	1989	28	Northern Ireland, Gulf War	Army	Military family, homesick when joining Army. Left Army involuntarily on medical discharge to work in the private sector. Attempted suicide due to pain from non-combat leg injury sustained in Gulf. Mobility somewhat limited and no present psychological needs. Volunteers in local veterans' project.
Andy	43	1989, 18	1997	7	Cyprus, Balkans	RAF	Sent away to boarding school, father worked away. Recalls good family relationship, but distant from father. Left Army voluntarily to work in private sector after 7 years with no medical. Employed 2 weeks after leaving RAF. Divorced, living alone for several years experienced depressive symptoms. Remarried. Slight physical disability.
Daryl	40	1994, 19	2000	10	Balkans	Army	Sent away to day boarding. Good family relationships. Operational tours of Bosnia (1996) and Kosovo (1998). Left Army voluntarily to work in private sector. Employed shortly after leaving Army. No psychological symptoms. Slight physical pain reported after jumping off a roof (accident). No affiliation to veterans' groups.
Mark	47	1983, 16	1989	6	Post-Falklands	Army	Good childhood family relationships. Joined the Army and left at rank of Sergeant Major. Stationed in Germany. Left Army voluntarily to work in private sector. Experienced psychological distress after divorce in 2005. Met new partner 2009. Slight physical pain reported in leg (accident) and became dependent on pain medication. Attempting to rejoin at time of interview
Lynda	43	1996, 25	2014	18	Balkans	Army	Emotionally abusive father, mother mental health issues. Left at rank of Lance Corporal. Stationed in Balkans then received surgery in 1990 for male-to-female transition. Left Army twice due to psychological issues experienced after suicide of mother, then brother.
John	43	1987, 16	2006	19	Northern Ireland, Afghanistan, Iraq	Army	No positive family relationships, no contact with mother, sister by choice. Deployed on six tours of Northern Ireland, five Iraq tours, and two Afghanistan tours immediately after 9/11. Divorced and remarried. Service injuries from shrapnel, stress reaction when return home. No current medical or psychological treatment.
Barry	56	1977, 19	1993	16	Cyprus, Northern Ireland	Army	Recalls positive family relationships. Boarding school, then joined the Army as an Officer. Deployed to Cyprus and two tours of Northern Ireland, married after second tour of Northern Ireland. Employed in private sector. No past or current medical or psychological treatment.
Morris	57	1974, 17	2012	38	Northern Ireland, South America, Iraq	Army	Distant relationship with father, but maintained contact with mother. Retired from Army after 38 years. Multiple operational tours to Northern Ireland. Suicide attempt after leaving voluntarily. Volunteer for local veteran charity.
Lionel	28	2004, 18	2010	6	Iraq	Army	At least one supportive family member, a good relationship with a successful uncle involved in his childhood provided alternative model of achievement. Joined Army as an Officer and left as a Commanding Officer after 6 years of service to work in public sector. Experienced nightmares and tremors post Iraq. No medical or psychological treatment sought.
Percy	56	1975, 17	2011	36	Germany, Balkans, Afghanistan, Northern Ireland	RAF/Auxiliary	Recalls positive childhood experience. Retired from RAF after 36 years, Joined RAF Auxiliary, deployed to Bosnia in 1994 exposed to gunfire and IED accidents as medic. Diagnosed with PTSD in 2010.
Eddie	53	1980, 19	1989	9	Post-Falklands	Army	Recalls positive family experiences in childhood. More distant relationship with father absent with work, limited contact with sibling post-service. Joined the Army and left with rank of Sergeant. 1994 met girlfriend (later wife) before Germany deployment. Operational tours of Bosnia (1996) and Kosovo (1998). Left Army voluntarily to work in private sector. Slight physical pain reported. No affiliation to veterans' groups.
Simon	49	1982, 17	2005	23	Northern Ireland, Gulf, Balkans	Army	Large family and positive relationships after father left home, remained in contact with father. Difficulty in school. Deployed on tours of Northern Ireland, Persian Gulf and Balkans. He married at 22. Medically discharged. Reported severe PTSD, childhood abuse, physical health issues related to Gulf deployment.
Tina	57	1974, 17	1979	5	Post-Falklands, Northern Ireland	Army	Recalls good but distant relationship with family. Deployed on one tour of Northern Ireland at 17. Left voluntarily Stress reaction and depression after return home. No current physical or psychological disorder reported.
Stewart	50	1985, 21	2008	23	Post-Falklands, Gulf, Balkans, Caribbean, Northern Ireland	Royal Navy	Recalls good family relationships. Multiple tours to First Persian Gulf War, Kosovo, Serbia, Northern Ireland, Caribbean and classified operational piracy and narcotics tours. After surgery, left voluntarily in 2008 after 23 years to work in public sector for veterans' health. No current physical or psychological disorder reported.
Frank	66	1964, 16	1971	7	Post-WWII, non-combat	Royal Navy	Recalls good family relationships with birth family and stepfather. Non-combat tour of South Africa. Left service involuntarily due to death in family. Trained to become a fireman. Married 1974, no children. Full-time carer to wife. No current physical or psychological disorder reported.
Aaron	49	1987, 22	1994	7	Post-Falklands, Germany, North America, Caribbean, Northern Ireland, Asia	Army	Recalls good family relationships. Two tours of Northern Ireland and Hong Kong tours. Reported small arms attack on second Northern Ireland tour. Diagnosed with PTSD in 1997, Reported suicidal ideation. No health or psychological issues reported.
Roger	53	1977, 16	2011	35	Pre-Falklands, Northern Ireland, Gulf War, Balkans, Germany, North America, Falklands, Caribbean, Africa, Middle East	Army/RAF	Recalls good family relationships joined Army, then joined RAF and trained as a pilot. Non-op tours of Falklands, operational Gulf War, Bosnia and Iraq/Afghanistan. Left service voluntarily and trained for private sector while in service. No health or psychological issues reported.

### Study Design and Interview Protocol

This study explored how ex-service personnel perceived their experiences in military and civilian worlds, ways in which those worlds may be distinct, and their roles within a broader social culture outside of the military. The individual's biographical accounts of military experience is as important as making sense of what lived experiences represent to the individual (24, 25). Narrative analysis is appropriate for exploring the psychological link between the developing self and the essential role of story in understanding a self over the lifespan (26). Additionally, using a thematic method allows for researchers to explore texts, analyse consequences of actions in personal narratives (27), and identify stories as either sharing similar patterns or divergent themes (28). The researcher consulted Portsmouth ex-Armed Forces individuals from local veteran charities on the research design of the study and who were not participants. Researchers in the field of military psychology were also consulted to formulate gaps in knowledge about this veteran population. An interview guide (Table 2) and interview questions (Table 3) were developed to capture participant narratives. Interview questions were consistent throughout all participant interviews. Any modifications were implemented during interviews to prompt participants for clarification where description of events, language, or terminology were unclear.

**Table 2:** Research interview guide.

**Demographics**	**Education**	**Family**
Where did you grow up	Did you like school *I was bullied* *I loved going and I wanted a PhD afterward*	What were your parents like *I had to be the man of the house*
What was your neighborhood like *Everyone went, we didn't know what we were doing* *It was rough, we were* *sent away to work on* *farms*	Did you have access to school *I didn't go to school for 2 years*	Did you have family members/siblings, did they serve *I didn't know but I became a fusilier, just like my dad. He never talked about it (WWI)*
What was it like growing up in that village/city/county	School achievement *They told me I wouldn't amount to much*	Did you have any other family members who joined up
When did you decide to leave home *Seeing what my future* *was going to be like, I didn't want that*	What did you want to do after you left school	What was it like growing up in your home *I didn't have a childhood* *My mum was hit*

*Items in the semi-structured interview with exploratory questions reliant on the participants' own ability for interpretation during the interview (24, 25). Questions about the participant's life began the process, and included topics of education background, service deployment, and family, for example*.

**Table 3:** Researcher guide for interview questions.

**Pre-service experience**	**Researcher prompt for reasons for joining and transition into service**	**Service experience**	**Researcher prompt perception of life inside Armed Forces**	**Post-service experience**	**Researcher prompt transition out of service**
When did you join the Armed Forces?	Prompt for childhood experiences and motivation for joining Armed Forces	Can you tell me about your experiences in the military?		Can you tell me about your life after service?	Prompt for well-being, quality of life and mental health provision experience
Why did you decide to join?	(If not conscripted) Why did you decide to join?			Have you experienced any issues since leaving the service?	
				Would you use civilian services?	

Participants were recruited via purposive sampling to include as broad a range of military experiences as possible (29). Potential participants were invited to read about the research after presentations of the research aims at veteran events or meetings with relevant community groups, veteran associations, and through adverts in local newspapers. Flyers with research information and invitation for participation were distributed to veterans' charitable organizations and online recruitment was promoted through veteran social networking sites and forums. Participants may have been discharged or left the services for a variety of reasons including medical discharge or retirement from the military. Additionally, stigma around veterans' mental health issues are both influenced and contextualized by cultural attitudes toward the war in question (30). Therefore, participants of different rank, gender, age, service length, and war cohort were invited to participate. Participants were excluded if they were currently serving in the UK Armed Forces, who did not have the capacity to consent to participating in the study due to cognitive impairment or any other physical or pharmacological impairment, or who did not make contact with the researcher. The number of participants was restricted to 30 due to saturation in the breadth of participant characteristics and preliminary analysis of data (31).

Participants who read about the research in forums or attended presentations of the study, and who showed interest in participating in the study, received research participant information research booklets containing participant information, consent forms and pre-paid envelopes used to return signed consent forms. At interview, participants were reminded of the aims of the study, and were provided with a sample of interview questions to explore, for example, when participants joined the Armed Forces, reasons for leaving, or experiences of mental health services (Table 3). Participants were reminded of their rights to withdraw before and after interview. Semi-structured interviews were selected as the method to collect biographical data (32). Interviews were audio recorded and conducted in person at participant homes or in other locations of their choosing. Telephone and communication software, such as Skype (based on participant preference and geographical location) when in-person interviews could not be arranged. In addition to gathering potentially new information about UK veterans' lives through their narratives, verbatim transcription captured any nuance in language veterans used to recount their life experiences in their own words (33). After interviews were concluded, participants were provided with a debriefing sheet containing contact information for local and national veteran support organizations as well as mental health charities. The researcher offered to follow-up with research participants 24 h after the interview according to the individual's contact preference indicated in the participant consent form. Participant interviews were transcribed, read and re-read to familiarize the researcher with patterns (or divergent codes) within and across the data set prior to analysis (28).

### Analysis

Twenty-four audio recorded participant interviews were transcribed by the researcher and six by a transcription service observing The British Psychological Society governance on confidentiality, anonymity and non-disclosure of the content of research participant audio recordings (34). Once interview recordings were transcribed, coding of transcripts was completed following repeated reading of the transcripts, drawing out patterns in the data, and cross-tabulating potential codes for the development of sub- and superordinate themes created by KG. CW and KB then checked coding in samples of anonymised interview data to triangulate and audit possible alternative themes. This process effectively allowed for the comparison and refinement of codes to finalize narrative themes in the data (35). This inductive process allowed for linking of themes grounded in the interview data and followed by in-depth analysis across the main narratives in the 30 interviews conducted (36).

The stories told by participants are complex human accounts of experience and qualitative approaches to collecting and analyzing interviews helps to organize and clarify the story (37). Experiences across the lifespan were further analyzed using Narrative Analysis to develop themes from codes and explore meanings participants made of pre- and post-service life, service in the Armed Forces and interpretations made between those experiences (37). NViVo 10 software was used to reduce duplication of codes that may occur during qualitative data analysis, particularly with long interviews (38). Using a thematic approach to access oral or written content, researchers can answer questions about unexplored perceptions and perspectives of a particular group “in relation to the broader social context” [(28), p. 93]. Combining qualitative methodological approaches for data analysis helps to deepen understanding of participants' interview data, thereby meeting the objectives of this study (39). Lal et al. (39) suggest combining analytical methods that complement each other. This allows for a thorough exploration of the construction and adaptation of story significant for explanations or representations of self.

The participants' meaning making process is important for the interview (particularly as part of data analysis) and the participant's ability to examine their lives derived from that evaluation. This includes developing positive narratives of post-service life (e.g., participants talked about their achievements in service). Extracts from the interview data illustrate the overarching themes. The notes made after the interview documented affective moments in the interview which audio devices could not record, and which aided the researchers' interpretation of participant accounts. Higate and Cameron (40) discussed reflexivity in the researcher's process particularly with regards retrospective participant interviews. Researcher reflections on each individual interview were recorded as each interview progressed to acknowledge and address potential bias and rigor in interpretation (41).

### Ethical Considerations

The study proposal, peer reviewed research protocol and ethical application, received a favorable ethical opinion from the University of Portsmouth Science Faculty Ethics Committee. Participants received information about the study in a participant research booklet containing a participant information sheet, with details of the research study, inclusion criteria, right to withdrawal, informed consent and use of participant data for dissemination Interviews were scheduled at least 24 h after the participant received the participant research booklet to allow sufficient “cooling off time.” The study did not involve human tissue use, or groups considered vulnerable by the definitions set out by the relevant authorities (such as the National Health Service, the Social Care Research organization, or the University of Portsmouth).

## Results

The analysis of recorded interviews developed from participants' stories about pathways into and out of the military, and life after service. Superordinate themes were categorized as follows: *reasons for leaving the Armed Forces, outside the military “bubble,”* and finally *mental health concerns after service*. The ways in which these constructs and related sub-themes contributed to narratives about post-service adaption, change in needs related to change of identity and military experience, and perspectives on mental health. Patterns identified in latent themes in the data will be supported by the participants' making sense of experiences (28). Subordinate themes ranged from both avoidance of negative memories and sense-making of military experience, to the lifetime impact upon the veterans' identity and on their peer and family networks. Finally, the stories that follow are not only about experiences after service, but also before and during service as they link to concepts of transition over the lifespan, and related perceptions of well-being.

### Reasons for Leaving the Armed Forces

The following accounts from participants represent key decisions to leave service and return to civilian life, which also created challenges for the individual and conflicts in the family. Participants described experiences of being sent into combat, long operational tours away from family (including non-combat experience), or disillusionment with military service life in general in terms of voluntary and involuntary pathways out of service.

### Military Experience No Longer Meaningful

*Military experience no longer meaningful* refers to one of several reasons ex-service personnel cite among their motivations for leaving. First, ex-service personnel discussed feeling underappreciated for their practical experience. Work in the service was reduced to following pointless orders and performing menial tasks. Second, participants also gave accounts of feeling disillusioned with the Armed Forces because they felt devalued by the institution itself. Third, participants expressed beliefs that the civilian population neither understood nor cared about their personal military experiences.

Daryl had talked about the long-term effects of consecutive tours of duty, and the negative impact on his family were central to the participant's motivation for leaving (see Table 1 for brief participant biographies). Not only was the participant relating his experience of being away from his family to a negative impact on the family, but he also compared his own childhood experience in which his father's absence had negatively affected him:

*so it was literally [emphasises each month by tapping table] six months, six months, six months, six months and-and new married life erm, didn't really want to go into-certainly didn't want to have a family in that environment-didn't want to do that ‘em, I wanted to be around wanted to be there* (Daryl, p. 47).

Other participants who had not planned a career in the military ‘*had a plan to leave*'… and “*would stop when* [she] *stopped enjoying it'* (Betty, p. 19). This participant enjoyed the uniqueness of working on a project within her field that matched her training and would utilize her skills. Planning a pathway out pre- provided a viable enough rationale to leave the Army and there was “*no reluctance at all to leave…because I had this massive goal- no-one in* [my field]…*was working on such a Gucci, um, exciting project…so it was a no-brainer”* (Betty, p. 9). Of the 30 participants interviewed, there were few ex-servicemen and women who were able to transfer their military skills to a role in the civilian world, or chose to leave voluntarily, or had a plan to leave *at the start* of their service careers.

Changes in the Armed Forces itself also caused participants to question the value the Armed Forces placed on service experience. Not being taken seriously for the experience that they had accumulated, conflicted with what participants wanted out of their service careers. Not being utilized properly for one's experiences and skills, lead to questioning one's purpose. Ex-service personnel organized their worldview from a position of being always available, ready to act under command, and to protect (like “*Supermen,”* Freddie, p. 4). The difficulty of moving on from one role in service with identifiable qualities, to another, less heroic and less defined identity post-service, was frightening. This sense of fear and unfamiliarity of purpose was explicit in the narratives of Jack, Martin, and Matty, and best summarized by Freddie:

“*I don't think you ever stop being a soldier. If you've done like 13 years, it's it's you know, it's a big part of your life, so I don't think it ever leaves you become indoctrinated. It's like, you know, prisoners who do 13 years a prisoner is scared of they're scared of being released and I guess it's similar for military guys. Some military guys.”* (Freddie, p. 4)

The Army, and being a soldier and specifically a combat solider, defined part of this veteran's life that he compared with being incarcerated. He likened his experience to repeat offending by individuals who once inside the prison system were scared and did not know how to cope with being outside of the military environment after leaving it.

### Developing Identities Through Military Service

Abuse or neglect pre-service are notably higher in veterans who have been exposed to higher levels of childhood adversity than non-service personnel—and may be under reported—which may prompt veterans to leave abusive home lives for the service (42, 43). Leaving service as a Lieutenant Colonel, with a “crown and star” on his shoulder Morris felt he did not have anything of importance to contribute from his military service and to the greater society he was about to enter (p. 16). Furthermore, Morris also experienced an existential loss, of disconnection from a life that had defined him:

*you end up with this sort of sense of belonging of course, but there's also a sense of doing something worthwhile and having control and having ability to influence things and all of a sudden the control I had was over…and what time I walked the dog, my ability to influence things was minimal rather than you know around the house sort of thing and it just uh. that sort of sense of self-importance*. (Morris, p. 17)

Becoming a veteran means no longer being able to identify with an organization that rewards unconditional duty and loyal service. However, challenges to one's identity occurs when participants “*fight the transition process”* (Morris, p. 19) especially when the transition is from a defined, valued and valuable role, into the unknown. The process of change is less disciplined, and far more unpredictable.

### “*Disabled for Life”*: The End of the Armed Forces Career

Fighting against or accepting the transition process featured heavily in narratives of participants who were deemed psychologically and/or physically incapable of fulfilling their duty. Leaving as a result of disability in service, is described by Nicholas and the frustration he experienced at not being able to rejoin his Army regiment: “*And in short, that's where I finished up, operated on my spine that was in'45. I have to say I was very upset then I had never got back to my regiment after'44”* (Nicholas, p.17). For Nicholas, his service career was finished through injury and not by choice. What he had experienced as a once young and “*fairly fit”* young man (p. 17) was now gone through a back injury that had kept him in hospital for a year. Nicholas “*never got back”* to what he was:

“*it's such a serious thing that that um head and spine you you've not much chance you going back to the Army as they say you're not fit enough to be a soldier and it-that-that…I thought that really hurt I thought ‘ugh' but there we are…I was just a young roe lad of 18 when I went and I came back all disabled for life.”* (Nicholas, p. 19)

Participants shared accounts of how they were coping with physical and psychological disability. After losing mobility in his lower back and shoulders, Freddie eventually went to the hospital in 2010 where he learned he had fractured his back as early as 1996 “*to 2010 [and] was running around with a broken back. Sounds silly, don't it?…That's what it's all about. That's why it's called ‘soldiering on'. Soldier on…You just keep going”* (Freddie, p. 29). Despite his body being compromised by the years and hardship of service training and repeated deployment to combat tours in Northern Ireland, Afghanistan, and Iraq, Freddie took pride in how he coped with his injuries. He acknowledged that as much as he regrets no longer being able to serve, had he remained in the military, his life might have been much more different: “*so the chances are if I'd have stayed in, I'd probably be paralyzed now from the chest down. So gotta you know gotta be grateful for small mercies, haven't ya?”* (Freddie, p. 32). Freddie had faced his own death and the death of others, survived close encounters with combatants, and yet wanted to continue to serve. He had delayed seeking mental health support while in service and post-service. However, when his body could not fight to overcome his physical injuries, preventing normal physical functioning, had to save his own life by cutting off his connection to the Armed Forces and from being a soldier. He could no longer “*soldier on.”*

Other participants described their experiences of involuntary leaving and the subsequent change in their identities as no longer military men and women. They struggled with leaving the Armed Forces and returning home, entering a new environment outside the safety of their military surroundings. The following section will explore participants' accounts and perceptions of good adaption and troubled transition from military service into the civilian world.

### Outside the Military “Bubble”

Some participants had trouble adapting to post-service life. In these participants' stories, specific skills acquired in service (sometimes developed over long periods of time) were now incompatible with or lacking direct transfer to civilian employment. Leaving was harder for those participants who longed to stay in service and were forced out. For example, involuntary redundancy was cited as either having an immediate or delayed impact on adaption after service. Delays in divestment of service members' transferable skills can be detrimental to overall veterans' sense of self-worth near the end of service life, and reintegration post-service (44, 45).

Troubled adaption was also linked to involuntary transition out of service when participants reported being disabled out of service through injury sustained in service or on operational tours. For veterans in the study, particularly WWII veteran Nicholas, “*civvy street, for the main part, [was] very difficult” and “the memory of this time is always there”* (Nicholas, p. 21). For certain participants, the magnitude of leaving the service created identity crises, resulting in feelings of exclusion and painful separation, or, an unwillingness to connect with the past reminders of one's pre-service life. The Armed Forces is seen and experienced by some, as a family. This type of family offers reassurance and protection in the form of commonality of purpose, unity, and togetherness with others (46). Once leaving the Armed Forces, a once protective and omnipresent bubble, bursts.

For Betty, on the other hand, being part of a group outside the service provided valuable informal support during her attempts to start a family:

[T]*here was an online help group and we used to meet up, well I met them a couple of times, so we knew exactly what I was going through. Brilliant, it was the best thing I did at that time, urm, yeah it was great…because I realised I was sad*. (Betty, p. 11)

This was perhaps the timeliest kind of help for this participant, needed at a difficult time and provided *outside the military “bubble.”* This participants' narrative pattern did not fit similar transition pathways into or out of service. Betty's contentment with her childhood environment and service life histories, and preparation to leave despite personal challenges, was a model of a relatively positive transition story compared to other participant narratives of well-being linked to pre-service and post-service life.

### Thriving in Post-service Life

Positive adaption was experienced when participants recalled little or no pre-service adversity, who prepared to leave the Armed Forces voluntarily, who had prepared financially for resettling, and/or formed and maintained friendships outside of military service with friends they could go to for support. Participants developed service skills that were easily transferred into civilian employment which aided transition.

Freddie felt that he had a good experience of the civilian world through his employment experience after service. The participant was earning more than he had as a soldier and has been promoted. But his work colleagues were predominantly ex-military personnel:

*I was working as a security guard in London erm there were er I was working for a company that sub-contracted…erm and it was the most boring job, but they wanted ex-military guys…and I got promoted a couple times quite quickly because they liked me* (Freddie, p. 25).

Freddie attributed a good working experience and adaption to civilian life to his ability to advance and earn more than he had as a soldier, transferring his combat skills to corporate security, and within an environment of ex-soldiers. Daryl did not have a difficult transition into civilian life and found employment as soon as he left the Army. His story is shared by few participants (like Paul, Roger, and Betty for instance). As he “*progressed in his career,”* Daryl found it “*very strange that people find it [civilian life] alien”* (p. 46).

Curtis had felt unprepared for post-service work. He reflects back on joining the Army as a time when he would have welcomed someone encouraging him to think about leaving and making a transition plan because leaving (for a variety of reasons) was inevitable: “*All I wanted…is someone to say to me: You might leave tomorrow and you've failed. You might get injured, you might leave for personal reasons, you might serve a full career, but you will leave”* (Curtis, p. 6). Curtis may have felt more in control of his future if at some point he had realized that he was not going to be a soldier indefinitely. Putting a plan in place toward making the adaption to civilian life a more positive experience, regardless of how long one's service career was, would aid more positive transition out of service.

Participants also experienced good adaption when it was their choice to leave service either through retiring or by not renewing military service contracts. Stewart made the decision that: “*at some point*, [he] *was going to have to make a second career. I wasn't gonna necessarily retire age 55 sit down and do nothing”* (p. 22). For Stewart, preparing to leave made transition a good experience that he looked forward to: “*transition from military to civilian was…an easy transition– for other people it wasn't as easy because they hadn't thought about ever leaving”* (Stewart, p. 22). Regrets about preparing to leave, was not distinguished by pre-service adversity, mental health challenges in service, or rank upon leaving the Armed Forces.

Barry had formed friendships outside of the military and this, he felt, helped him to remain connected to a civilian world outside the Armed Forces. Stewart also associated good adaption with his and his wife's network of friends outside of the military who were sources of support. Support was forthcoming during service and even after the participant left. The individuals who share this narrative struggled with leaving the service at first, but eventually thrive in civilian life. Few participants reported this pattern. Having a supportive network outside of the Armed Forces in pre-service family life and after service, is a narrative shared by those *thriving outside the military bubble*.

Eight participants whose narratives involved feelings of abandonment by family of origin (through neglect or actually leaving the participant), join the Armed Forces which becomes a substitute for family. Curtis' view was that for some “*the Armed Forces, particularly in the early years of someone's career, are in loco parentis, particularly w-when the person who's joined, is young*” (Curtis, p. 6). Participants' attachment to a substitute military family created a sense of stability, experiences of more secure attachment and coherent narratives in adult relationships after service (47). Seven of those eight participants also shared a re-abandonment narrative: abandoned by family pre-service, abandoned by military post-service. This was consistent even with participants who were not at risk of exposure to service trauma, but experienced abandonment from an abusive family or the military family. Understanding narratives of veterans who both strive and struggle, can inform researchers about specific difficulties veterans are experiencing that may not be linked exclusively to the impact of service history.

### Narrative Meaning-Making and Evaluation of Life After Service

As in positive adaption to post-service life, this theme references the continuation of family heritages, pride in the family, assessing challenges and how they were overcome (pre-during and post-service). Paul talks about his own son, growing respect for his son, and his son's decision to join the service:

…*a lot of people have asked me the question: Well is it because he's joined the army and he's followed in your footsteps, that you've started to respect him more? And I thought well, yes, there's a lot to be said for that* (Paul, p. 48).

When participants talked about their achievements in their post-service lives, they talked about the achievements of self through family, which were exemplified by feelings of success or pride in family members making a “*success of their lives”* (Nicholas, p. 27). One's satisfaction in overcoming the hardship of war, or personal obstacles of being considered “*not very bright”* (p. 24), culminated in a “*brilliant”* life for the 92 year-old WWII veteran participant (Nicholas, p. 22–27). Freddie evaluated his life as being more important after service; to be available to one's family which meant being “*emotionally available”* when his own children needed him, unlike his distant father.

Putting one's family above one's own psychological challenges meant not getting “*stuck”* in the consequences of ruminating about early childhood adverse experiences:

*Yer just gonna end up not being able to look after yer family and look after yerself. And my family's the most important thing to me. Doesn't matter what happens to me as long as they're alright and as long as they're looked after, that's fine* (Nicholas, p. 34).

The participant wanted to take care of his own family regardless of what might happen to him. Resisting the reflexive process that may occur in the therapeutic setting, also served to meet the needs of putting family first, at the cost of taking care of one's own well-being needs (48). Six other participant interviews revealed a similar theme about making sacrifices for family. This feeling was shared by participants regardless of whether they had suffered adversity in childhood, felt disconnected from family, or whether they voluntarily or involuntarily followed family tradition of joining the Armed Forces. Additionally, participants who had experienced difficulties pre-service and in the Armed Forces (e.g., John, Terry, and Simon), expressed support for their own children joining the military, knowing of the potential challenges and sacrifices.

Freddie reflected positively on the service life he gained from being in the Royal Marines: “*the military made me who I am now and I think I'm a better person for it”* (p. 32). An integrated sense of self, separate from how one defines self through career, is necessary for a feeling of satisfaction and thinking positively of the future (49). Mental health concerns and experiences of mental healthcare and support post-service were explored to understand to what degree (if any) participants linked post-service experiences to their current well-being and whether pre- or service events are integral to those perspectives which create conflicts in overall sense of well-being and coping with change after leaving the Armed Forces.

### Mental Health Concerns After Service

Some veterans experience mental health difficulties while in service, but only report problems at some point after they leave the service [for example (50)]. All research participants were asked if they sought or received some form of psychological help, even if they had not reported experiencing mental health problems while in active service. They discussed mental health challenges, overcoming difficulties after leaving, and issues encountered by family as a result of mental health concerns.

### Accessing Health and Well-Being Services

This theme includes types of mental health concerns, as well as access (and barriers) to psychological and social (practical) services. The lack of formal services or limited knowledge about what formal services are available is experienced by and influences veterans and their families' sense of well-being post-service. Participants tended to talk predominantly about their experiences with mental health services, but they also occasionally included accounts of the effects on the family when experiences of mental and other types of well-being services were evaluated.

John and Freddie provided experiences about the loss of excitement they had received from being in combat. For Curtis and Aaron, avoiding being killed in Afghanistan and Northern Ireland, respectively, gave them a level of excitement. However, when those periods of activity were replaced with inactivity, participants recalled their sleep being disrupted or disturbed:

*Apparently I twitch in my sleep, and I never used to twitch. Ever since I came back, I shake in my sleep…it doesn't affect me in any way, but it sometimes just keeps [my wife] awake…and she worries about me. But yeah, so she-so I twitch now in my sleep which I don't-I don't know if it's related, but she said it's ever since I got back (Curtis, p. 65)*.

Participants like Curtis, Aaron, and Lionel, for example, reported troubled sleep. Lionel's involuntary physical tics began after his return from Afghanistan and occurred at night (such as sleepwalking for Curtis). This was perhaps a physical manifestation of trauma, and an analysis of his reactions could be linked to a normal reaction to being exposed to combat, which also affected his partner (51). For Aaron, Jack, and Morris, a silence that follows along with inaction and loss of purpose gives way to memories of attack and danger. However, not accessing services and barriers to getting help were experienced among ex-service personnel, when

*sitting in front of a counsellor and the counsellor with the best will in the world and experience – they don't understand what that's soldiers' talking about, they'll get up and walk out, because they'll know that you don't understand what they're talking about* (Paul, p. 20).

Paul analyzed a period in his life where if he had “*attempted [suicide] and woken up and still been in this pain*, [he] *would still have attempted it”* and to prevent further suicide attempts would require psychiatric treatment or “*to put me somewhere”* (Paul, p. 30). Witnessing this pattern in other ex-service men and women, according to Daryl (while stationed in Kosovo), points out the barriers and stigma that renders a sense of helplessness: “[W]*hen I think back and quite embarrassed to say I wasn't supportive at all but-but, I don't know if it was the environment and it was expected of you, but it was seen as a weakness”* (p. 43–44).

Asking for help for mental health problems would be seen as a sign of weakness while on active duty. But Daryl's recall was one of regret after service for not offering or being able to provide support to someone in his Army unit who needed his help. Participants realized they were having problems coping, but formal support was thought of as hard to find, not available, not offered or denied to veteran and family members alike. Even, for example practical information about social services. These barriers existed for earlier WWII veteran cohorts. Support, as interpreted by this veteran, was a labyrinthine task: “*Things [resettlement information] are there but they're filed away, squirrelled away down stovepipes and rabbit holes, and the language used isn't helpful and it's all there, but you need a guide and an interpreter. So that-that's clearly wrong'* (Curtis, p. 6).

Betty's was one of the few veteran stories of positive experiences with mental health care. There are a few stories of ex-service personnel who found their voices and asked for help, and their perceptions of mental health help once received was positive. Betty described accessing formal support from a charity specifically tailored to veterans' needs, “*at any time if we have any issues” and over any length of time (“could be 18 months ago when it was really bad”) as and when needed* (Betty, p. 16).

### Rejecting Mental Health Symptoms or Diagnosis

Adding to the issues around access to mental health and well-being services, is the theme of *rejecting mental health symptoms or diagnosis*. Participant stories in this theme centered on patterns of noticing a change in their behavior or self, then concern. Participants who sought help, either thought diagnosis was helpful because the problem was identified, or, they rejected any mental health diagnosis.

When John first reported having mental health problems, he gave an account of being diagnosed with battle stress. Wanting to learn more about why he was stressed out and not able to go on combat tours, he recalls searching for and rejecting his own symptoms of PTSD, stating:

*that's what I thought I had, cause obviously I've killed people and done stuff so I was like ‘Oh I'll put that in' and I thought it's not that, I'm not having suicidal thoughts and what have you…I ruled that out straight away'* (John, p. 34).

Because casualties in combat was an obvious condition of his work, John had ruled out killing combatants as having any potential traumatic impact on his behavior Terry, Jack, Martin and Curtis all shared this reality of their military experience. But as Terry reports*: “nothing prepares you for killing”* (Terry, p. 10). Not being prepared for how his role in the Armed Forces would affect him, suffering from mental health problems as a result of service, and initially rejecting the cause of mental health difficulties, was a feature across six of the participants who had experienced combat. One participant who experienced combat in Northern Ireland, however, felt that this experience may have affected them, but did not wish to seek mental health care for fear of “*what I might find”* (Tina, p. 46). Additionally, two participants reported experiencing mental health problems, but had no combat experience, no direct threat to life in service, who also rejected their diagnoses. Having experienced the height of violence in Northern Ireland as a teenager in the Army, Tina was aware of what may have been a service-related psychological issue, however the participant feared “*wandering off into my Pandora's box that's been locked for a veeery long time”* (Tina, p. 46), and revisiting those experiences would be detrimental to well-being and everyday functioning. Suppression of troubling thoughts was easier “*because if you open it up, I would be gone. I'd be needing [psychiatric] services, not a psychologist”* (Tina, p. 61).

Unlike Nicholas and Derrick who did not have the option of formal healthcare during and immediately after WWII, post-WWII veterans who were provided public healthcare (and mental healthcare) perceived the National Health Service (NHS) community mental healthcare services, as inaccessible or uninviting. Evaluation of mental health provision was vividly described by Aaron who said: “*talking didn't help. The thing that didn't help [was] talking to someone who had no experience. The things that made me safe, were my bulletproof vest…Counselling was pointless, and so upsetting. Just didn't seem to work”* (Aaron, p. 19).

One ear after leaving the Armed Forces, Freddie was still experiencing mental health problems. At the advice of a friend, the participant contacted a veteran's mental health charity for help however he decided not to receive help under residential care because “*they basically wanted me to come live in for a week and I said: no, I'm not-I'm not doing that. I'll deal with it myself. So I did”* (Freddie, p. 25). By normalizing his experience, Freddie felt that he was like everybody else, comparing his occasional nightmares to “*bad dreams”* that people have (Freddie, p. 26). He reiterated he did not have PTSD, that he “*just needed time to process everything”* (Freddie, p. 26). Freddie would, however, seek formal medical help for physical injuries he sustained while in service. Seeking help for specific physical injuries as a result of service appeared to be more commonplace for veterans in this study, however there are still perceived barriers in asking for help even when unrelated to psychological well-being, and even when family members recognize the need for help, veterans avoid treatment, preferring in some cases to avoid well-being concerns or “*deal with it”* on their own.

## Discussion

Teachman (52) and Walker (53) found that few opportunities have been taken to adopt a lifespan perspective to explore the whole storied life of ex-service personnel and the experiences that veterans believe may have impacted them. By looking at stories people tell about themselves over an individual life course, researchers can understand how people behave, what motivates behavior, and what personalities people choose to become and how their identity is developed and maintained (54). Additionally, mental health evaluation occurs during a time of transition and at varied points in the veterans' life. A barrier to getting or accepting an offer of help presents a conflict between diagnosis and military identity, which reinforces stigma around help-seeking (55). Sub-themes of accessing well-being services or rejecting diagnosis are experiences of military personnel that are seldom understood. Furthermore, we know little about the coping strategies or help-seeking behavior of partners of veterans who report experiencing secondary trauma as a result of veterans' exposure to service-related events (18, 51, 56, 57).

The sub-theme *narrative meaning-making and evaluation of life after service*, links into the reasons for leaving the Armed Forces, the challenges encountered, as well as the concerns about mental health which affects the individual as well as the service member and their family networks (58). Leaving and transitioning are a constant part of many veterans' life stories (59). Upon leaving the Armed Forces and becoming veterans, they can no longer access original familial social networks for social support, access to service life is cut off, making navigating civilian networks difficult for veterans and their families (60). The transition from one's childhood family to becoming a military individual, then adopting new civilian identities (in late adulthood for some veterans), brings the researcher closer to the participant's reality and veterans' experience of exclusion (9, 43). Veterans either avoid narratives of war, or they make meaning from the extraordinary events of those war experiences which influences level of interaction with their civilian world (61, 62). In doing so, reasons for leaving the Armed Forces, changes the meaning that military service has for participants. On reflection, service life (and particularly the stories of returning from deployment), were similar to being caught in the past, feeling as if one was behind the rest of the world, or distant from society. Participants had been out of contact with family (voluntarily in some cases). Talk of service experience (particularly in combat conditions) is avoided with intimate partners and friends, and family members (63). Avoiding or not being able to access help as and when needed, links the stories of these participants. Participants can feel connected to their surroundings if they are still able to engage with and contribute in some way to the world while they are still in service and to continue valuing veterans' contributions when they transition out of service (64). Through sharing these narratives in non-clinical settings, veterans can influence interventions that may support them not only psychologically, but in social situations and for practical purposes (65).

There was a difference between the value placed on learning and skills acquired in service and skills of civilians in comparative fields of work. Participants found that there was no acknowledgment of what they had achieved and more importantly, little or no transferability of skills developed in service. The findings of the earlier FiMT 2013 survey on UK veterans proposed that the “military provide significant provision for transition on leaving the forces, more than any other employer” [(66), p. 36]. However, the demands of the type of military employment common to military veterans, and the conditions of that employment are not comparative with civilian employment. Nor is it comparative to transition out of the workforce, particularly when compared in the context of wartime demands on personnel. Participants experienced feelings of inadequacy, a loss of feeling meaningful, brought on by an uncertainty with one's role (67, 68). Being outside the military bubble symbolized vulnerability for those unprepared for transition, or an opportunity to identify adaption as successful, establish new narratives about roles, contributions and value to a civilian world (11). The provision of *significant* tools for transitioning were alternately experienced as non-existent, difficult to access or poor for those participants who had no choice when leaving the Armed Forces involuntarily, or provisions were experienced as good transition for those participants who left voluntarily and had planned to leave. Stories of thriving outside of or being challenged by post-service life, framed participant accounts on both sides of the military shell (69). The ability to incorporate a story of one's personal and professional identity, and form a dialogical narrative of self, is a prerequisite for this process (70, 71).

Also important to note is that participants who left in higher ranks, or began as officers generally had positive experiences of transition (1). Contrasted with those experiences, are the accounts of participants (young and old) affected by impoverished childhoods. The opportunity to join the British Armed Forces presents an opportunity for not only survival, but economic mobility (12). However, transitioning out of service without support is experienced as re-abandonment, particularly when external familial support was lacking to begin with. One participant essentially rejoined the Armed Forces on two occasions and therefore, did not experience a typical transition event, because external support was insufficient. Very few studies had looked extensively at the experiences of transition and resettlement issues for UK veterans and their families before the Armed Forces Covenant was published (66). Fewer studies consider the role of the veterans' childhood family experiences as potentially contributing to transition issues (6).

Participants described having to learn how to navigate a new environment outside the military, whose social norms and practices were unfamiliar. This may lead to troubled adaption in civilian life because the military community is no longer accessible and the discipline and skills learned to become part of a cohesive military unit are no longer useful for making connections with others in the civilian world (16). Trivializing exposure to surviving Bosnia, for example, or repeated tours of Northern Ireland and Middle East conflicts, and what learning was gained from these tours, were slowly becoming devalued inside service as well as by civilian employers and non-military colleagues alike (72–74).

Participants' own self-diagnosis occurred where they did not see the problem or, not having a concept of good psychological well-being, participants were unable to compare good against poor psychological health and avoided care of any kind (75, 76). In some participants (and consistent with the research literature), there were immediate or delayed reactions to service-related mental health problems complicated by level of avoidance or rejection of symptoms (50, 76). In narratives where participants were content with their lives, and experienced good transition, the family of origin creates a happy childhood. Military service is seen as an adventure, resulting in no mental health problems. This participant was satisfied with civilian life. However, narratives of positive transition to civilian life are not often covered as extensively in the research literature. The influence of childhood trauma experiences such as witnessing the physical assault of a parent affects physical, psychological, social well-being after service, and impacts on veterans' help seeking behavior (10), and over the lifecourse (43, 77). It is also important to acknowledge narratives of individuals who are able to overcome difficulties in their military lives perhaps as a result of their developing robust well-being from childhood, developing their own positive growth narratives (14). Participants who experienced growth from and coped with their experiences also recognized when support was needed, the type of support required, and that support was available (78, 79). In some cases, participants who shared similar accounts of positive childhood experiences, make conscious decisions to transition out rather than expose their families to the long-term impact of their military careers (66).

## Limitations and Future Research

More questions that the analysis of participant data raised were related to the differences and similarities that could be explored between young and old veteran cohort stories (80). An interpretation of the interview data tentatively suggests that for some service personnel who had experienced adversity or may have been vulnerable to delinquency in early life. A link can be made between choosing a military service experience and having a positive outlook on one's lifecourse regardless of whether the individual served in WWII, Bosnia, or Iraq for instance. Would other cohorts tell different stories about early life experiences and how divergent would they be? Would cohorts share similar long-term mental health and well-being outcomes if those veteran populations report similar adversity and resilience experiences of childhood? Veterans unwilling to communicate with civilians and/or family members about the psychological and physical struggles they encounter, obfuscates the desire or will to ask for help, occasionally leading to self-harm, such as those experiences shared by veterans of the Falklands and Gulf Wars (55, 81). Perhaps future research in the area of lifespan studies of veterans could explore the difference in UK ex-Armed Forces cohorts if meaning is made in different ways about the individual's life and how cohorts make meaning differently (63, 82). The researchers maintained integrity of the analytical process by remaining close to participant data as the source of rich theme development. However, an audit of the analytical process by independent researchers and the involvement of participants to validate theme development may have also provided another layer of rigor to the interpretative process (83).

## Conclusion

Mental health and well-being are linked to identity. The loss of identity as serviceman or woman both creates a vulnerability to mental health symptoms as well as exacerbating symptoms. This also leads to whether support is sought out or avoided. A tradition of family in service and perception of family were frequently referenced in participants' narratives about avoiding talk of war. If participants had been prepared for what to expect on deployment by family members who had military experience, they evaluated their own well-being based on how family members in the past had responded (or suppressed symptomatic responses) to war exposure. This study explored the ways in which ex-service personnel perceived their own well-being needs, particularly where participants' relationships (or lack thereof) with original family and own family may influence decisions to seek both formal and informal practical, social, and emotional support after leaving the Armed Forces. Responding to the growing interest in the social, psychological, political, and cultural dialogue about our veterans and their families' needs, insists on a review of mental health and well-being which also includes discussions of physical, social, and financial well-being with veterans and their families as essential to the research focus and outcomes. Highlighting the voices of veterans, raises awareness in the UK about the extraordinary as well as the everyday lives of veterans who are a part of our society.

## Data Availability Statement

The participant data collected and analyzed during this study are not publicly available as any personal identifying data was destroyed upon transcription of audio recordings. All transcribed data were anonymized at the point of transcription.

## Ethics Statement

The studies involving human participants were reviewed and approved by University of Portsmouth Science Faculty Ethics Committee. The patients/participants provided their written informed consent to participate in this study.

## Author Contributions

KG developed rationale and research method, designed study, collected data, audio recordings and transcription of audio recordings, analyzed data, and wrote each section of the journal manuscript. KB and CW reviewed and edited original draft and current manuscript introduction, methods, and sections of findings.

### Conflict of Interest

The authors declare that the research was conducted in the absence of any commercial or financial relationships that could be construed as a potential conflict of interest.
